# Coaching Bilingual Speech-Language Student Clinicians and Spanish-Speaking Caregivers to Use Culturally Adapted NDBI Techniques with Autistic Preschoolers

**DOI:** 10.3390/bs15091292

**Published:** 2025-09-22

**Authors:** Richelle McGuire, Jessica Nico, Naomi Nattress, Carlos Irizarry-Pérez, Cindy Gevarter

**Affiliations:** Department of Speech and Hearing Sciences, University of New Mexico, Albuquerque, NM 87106, USAjnico@unm.edu (J.N.); nnattress@unm.edu (N.N.); nyec@unm.edu (C.I.-P.)

**Keywords:** bilingual, autism, Spanish, naturalistic developmental behavioral intervention (NDBI), cascading coaching, culturally adapted

## Abstract

A cascading coaching model was used to teach bilingual speech-language pathology (SLP) graduate student clinicians and Spanish-speaking caregivers to implement naturalistic developmental behavioral intervention (NDBI) techniques with autistic preschoolers. Two triads (each consisting of a graduate student clinician, a minimally vocal child diagnosed with autism, and a caregiver) participated in the study. Following the cascading approach, a lead instructor (with limited Spanish conversational skills) coached bilingual student clinicians (in English) to apply culturally adapted NDBI with child participants. Following additional instruction in coaching, student clinicians coached caregivers in Spanish. Effects were evaluated using a multiple methods approach consisting of multiple probes across participants single case experimental design and a qualitative analysis of semi-structured interviews with adult participants. All adult participants increased their use of targeted NDBI skills including elicitation techniques (creating communication temptations, using wait time, and prompting) and response techniques (reinforcing children’s communication with natural consequences and providing a contextually relevant vocal model), demonstrating large to very large effect sizes. Although qualitative findings indicated areas for improvement (e.g., additional Spanish supports for clinicians), thematic analysis revealed additional benefits in terms of positive changes across adult learning, behavior, and perspectives; child communication; and child-caregiver relationships.

## 1. Introduction

Autism spectrum disorder (ASD) is a developmental disability characterized by social communication and interaction challenges, along with the presence of restricted or repetitive behaviors and interests (American Psychiatric Association, 2013). Around 30% of autistic^1^ children who use 0–30 spoken words are considered minimally verbal (Kasari et al., 2013). During early intervention, minimally verbal autistic children are often taught to use multimodal communication forms such as natural speech, gestures, signs, and aided augmentative alternative communication (AAC) systems. Unfortunately, in comparison to white non-Hispanic peers, autistic Latino^2^ children participate in early intervention at lower rates, receive fewer therapy hours/specialty services, and have higher unmet service needs (Guerrero & Sobotka, 2022; Magaña et al., 2013; Zuckerman et al., 2017). Additionally, most intervention research involves white, autistic children from monolingual English-speaking homes (Douglas et al., 2024; Steinbrenner et al., 2022). There is a critical need for research that explores how evidence-based interventions can be adapted to meet the needs of culturally and linguistically diverse (CLD) families, including Latino children from Spanish-speaking homes (Douglas et al., 2024).

Naturalistic developmental behavioral intervention (NDBI) is an evidence-based approach that can target multiple modes of communication for young minimally verbal autistic children and is adaptable for CLD families (Douglas et al., 2024; Pope et al., 2024). NDBI incorporates behavioral and social-developmental principles in everyday routines and contexts such as play. Communication partners incorporate elicitation techniques (e.g., communication temptations, time delay, and prompting) and response techniques (e.g., natural reinforcers for communication, speech models mapped to communication) within routines that included child-led interactions (Gallegos et al., 2024). Although NDBI is typically led by professionals such as speech-language pathologists (SLPs) or board-certified behavior analysts (BCBAs), it often includes caregiver coaching in which family members are taught to use NDBI during selected routines (Gallegos et al., 2024). Coaching promotes generalization of skills, and increases caregiver knowledge and empowerment (Lopez, 2024; Rieth et al., 2018). Although coaching involves families, adaption is needed to align intervention goals and methods with cultural values. When interventions values align with family values, positive outcomes are more likely; however, when values are culturally incompatible, failure to implement recommendations or service discontinuation may occur (DuBay et al., 2018; Long et al., 2015; Sands et al., 2023). 

Scoping reviews of cultural considerations in caregiver-led interventions and NDBI highlight a need for more research involving autistic children and families from a variety of cultural and linguistic backgrounds (Douglas et al., 2024; DuBay, 2022). Promisingly, a growing body of research has begun exploring culturally adapted NDBIs for Spanish-speaking families (e.g., Lopez, 2024; Meadan et al., 2020; Pickard et al., 2024). In prior research, an ecological validity framework or EVF (Bernal et al., 1995) has been applied to increase cultural sensitivity for Latino and Spanish-speaking families (Pickard et al., 2024; Sands et al., 2023). The EVF provides recommendations across different dimensions such as language, persons, and contexts (Buzhardt et al., 2016; Sands et al., 2023). Common EVF adaptations in NDBI studies have included providing all materials in Spanish and utilizing bilingual coaches (Meadan et al., 2020; Lopez, 2024; Pickard et al., 2024). Although culturally adapted NDBI studies have reported positive outcomes, limitations include a lack of data on caregivers’ experiences with NDBI (Lopez, 2024; Meadan et al., 2020; Pickard et al., 2024). 

Despite limited intervention research, qualitative research findings suggest that although Hispanic families generally endorse many components of NDBI, some techniques may be more difficult to implement than others due to conflicting cultural values (Cycyk et al., 2020; DuBay et al., 2018; Rollins et al., 2024). For example, in one qualitative study, Spanish-speaking parents of young children with ASD endorsed NBDI components such as following the child’s lead, physical prompting, and modeling (DuBay et al., 2018). However, the wait time and environmental arrangements were not as widely accepted because some parents were concerned that these strategies would frustrate their child (DuBay et al., 2018). While Rollins et al. (2024) also found that wait time may be a concern, contrasting with DuBay et al. (2018) their findings suggested that following a child’s lead may be challenging because it can be contrary to the cultural value of *respeto*, which emphasizes the importance of obedience, good behavior, and deference of children to adults, with interactions tending to be adult led. Findings from Cycyk et al. (2020) suggested a similar identification with adult-led approaches, as parents reported not being accustomed to modifying their communication to match children’s skills. Notably, Spanish-speaking parents reported that they would be willing to attempt difficult strategies if they felt the outcome would be worth the effort, and the clinician provided strong rationales (DuBay et al., 2018). Thus, to address potential cultural-mismatches, researchers must consider when to employ rationale building versus when to modify or adapt interventions.

Research exploring Spanish-speaking caregiver interactions with children with language delays also provides guidance regarding NBDI adaptations. For instance, Spanish-speaking families from lower SES backgrounds may use more directive adult-led interactions and spend less time narrating (i.e., describing and commenting on) children’s play in comparison to caregivers from non-Latino English-speaking high SES backgrounds (Peredo et al., 2020). Importantly, Spanish-speaking families also frequently use words of affection/affirmation and respond to children’s utterances (Peredo et al., 2020). Thus, a strengths-based intervention could involve teaching Spanish-speaking caregivers to notice and respond to child’s interests within adult led activities and respond to children’s communication with contextually relevant language (Peredo et al., 2018; Peredo et al., 2020).

Conceptually, many EVF adaptations could be easily incorporated in NDBI, however the availability of bilingual clinicians significantly impacts the use of language and person adaptations. Prior research has suggested that Hispanic families prefer having access to a bilingual, preferably Hispanic, coach rather than working with an interpreter (Buzhardt et al., 2016). Crucially, the availability of Hispanic, NDBI-trained bilingual clinicians is limited by the lack of diversity amongst SLPs and BCBAs. For example, only 8.6% of SLPs in the US identify as multilingual (ASHA, 2025). Of those that are multilingual, 67.7% indicate being Spanish-language providers, but only 48.3% identify as being Hispanic or Latino. Although data is not available on the language status of BCBAs, only 13.4% self-identify as Hispanic or Latino (BACB, 2025). Additionally, although bilingual SLPs often report being more confident in serving bilingual children than do monolingual SLPs, they also report being less confident in working with bilingual clients as compared to monolingual clients (Martinez-Nieto & Gilbertson, 2025; McKenna et al., 2025). Bilingual SLPs also report a variety of resources, knowledge, and training barriers at preprofessional and professional levels that impact their confidence (Martinez-Nieto & Gilbertson, 2025; McKenna et al., 2025). For instance, in one nationwide survey of bilingual and monolingual SLPs, less than half of participants reported learning methods to support bilingual children’s use of their home language during their graduate clinical education programs (McKenna et al., 2025).

One way to address these barriers as well as shortages of highly trained bilingual clinicians is to implement cascading models of coaching. In these approaches, an experienced clinician or researcher first provides instruction to less experienced clinicians who work regularly with caregivers, with the intention that the less experienced clinician will eventually implement the caregiver coaching themselves (Gallegos et al., 2024; Gevarter et al., 2021; Meadan et al., 2020). These programs incorporate research-based coaching methods including (a) joint planning (e.g., discussion of priorities, goals, and routines) and pre-session check-ins, (b) observing interactions between the caregiver and child during routines, (c) describing and modeling intervention techniques (supported with written content), (d) providing practice opportunities during routines while providing feedback/assistance, (e) engaging in post-interaction discussion/reflection; and (f) fading or removing coaching (Friedman et al., 2012; Gallegos et al., 2024; Minjarez et al., 2020b). In cascading programs, these coaching techniques are first employed by the researcher/experienced clinician to teach the less experienced clinician to use NDBI techniques with a child. Afterwards, the less experienced clinicians use the same coaching methods to teach caregivers to utilize the same NDBI techniques during routines with their child.

In a study by Gallegos et al. (2024), after receiving NDBI instruction and coaching from a BCBA-D, SLP and special education graduate students coached Hispanic, English-speaking parents of minimally verbal autistic children. Student clinicians were able to implement coaching techniques with high fidelity, and parents and students increased their use of targeted NDBI techniques. Caregiver and students reported positive outcomes on a post-intervention survey, however in-depth interviews were not conducted, and language adaptations were not deemed necessary. Compellingly, a cascading model may serve as a viable option for increasing Spanish-speaking families’ access to NDBI as experienced clinicians who lack the Spanish skills to coach parents can provide instruction/coaching to bilingual graduate clinicians who can coach parents. Additionally, applying a cross-linguistic cascading coaching model of instruction within a graduate training program provides a novel approach to addressing clinical education barriers reported by SLPs who work with bilingual clients (Martinez-Nieto & Gilbertson, 2025; McKenna et al., 2025). Thus, the purpose of study was to extend the findings of Gallegos et al. (2024) by utilizing the same cascading coaching approach to NDBI to facilitate coaching of bilingual graduate clinicians and Spanish-speaking caregivers of autistic children. The primary research questions included:

1. Does a culturally adapted cascading coaching model lead to an increase in bilingual student clinician and Spanish-speaking caregivers’ use of NDBI techniques?
2. What are caregiver and student clinician’s perspectives regarding the effectiveness of the program and areas for improvement?

## 2. Materials and Methods

### 2.1. Participants 

Participants included two triads that each consisted of (a) a minimally verbal autistic child from a Spanish-speaking home, (b) a participating Spanish-speaking caregiver of the child, and (c) a bilingual (English and Spanish) graduate student clinician. Children and caregivers were recruited via outreach to local early intervention agencies, preschools, private SLPs, and parent Facebook groups. Student clinicians were recruited from a group of SLP graduate students participating in an autism training grant and summer clinic.

Child participants met the following inclusion criteria: (a) aged 2.5–4.11 years old; (b) had an independent diagnosis of ASD further confirmed via the Childhood Autism Rating Scale second edition (CARS-2; Schopler et al., 2010) or the Spanish version of the Gilliam Autism Rating Scale Third Edition (GARS-3; Gilliam, 2014); (c) reported to use less than 30 spoken words (i.e., minimally verbal); (d) lived in a home where Spanish was the primary language, confirmed by caregiver report. Students and caregivers completed an approved consent process, and parents completed consent for children (which included information about child assent).

Table 1 outlines demographic and assessment information for child participants, Zandra and Gustavo (pseudonyms). With respect to each child’s communication skill repertoire, Gustavo primarily relied upon unconventional and conventional gestures to communicate (e.g., reaching, pointing) but his mother reported he had recently started using the sign *MORE* and the vocal words *No* and *Hi*. Zandra’s mother reported that her daughter primarily communicated via unconventional communication forms (reaching, leading, pushing away items, persistent non-word vocalizations) and would engage in challenging behaviors (e.g., screaming, eloping, self-injurious behaviors) whenever her mother tried to prompt her to imitate vocal words.

Table 2 outlines demographic information for adult participants (with pseudonyms). Participating monolingual Spanish-speaking caregivers (both born in Mexico) included Zandra’s mother, Fernanda, and Gustavo’s grandmother, Martha (a primary caretaker). Fernanda reported that she had participated in early intervention services with Zandra, and that Zandra was also currently receiving SLP services at school. Fernanda reported challenges with being able to access high-quality services for Zandra in her rural area and was on a waitlist for ABA. Gustavo’s mother reported that he had participated in early intervention services, but that his grandmother had not been a primary participant in the program.

Student clinicians included Nadia and Raquel, fluent English/Spanish speakers who identified as females of Mexican heritage. Although there were seven student clinicians participating in the summer clinic/training grant, excluding author 1, Nadia and Raquel were the only two clinicians who spoke Spanish. At the start of the study, both had completed two semesters of graduate work (including a course focused on social communication intervention for autism and developmental disabilities). During her undergraduate program, Nadia had worked in an elementary after school program that included autistic individuals and reported having an autistic cousin. Raquel had more extensive experience, working part-time as a registered behavior technician in an ABA clinic (that primarily employed discrete trial instruction methods) throughout her undergraduate program. Neither student had direct experience coaching caregivers.

#### Screening Assessments

During screening, the first author interviewed the mothers of child participants. Spanish assessments were used with Zandra’s mother. Both children were present during initial screenings and were engaged in play with a research assistant. Although parent report was the primary assessment technique, informal observation of the communication interactions between the child and research assistant were used to confirm or clarify parent report of communication skills. Although Gustavo’s monolingual Spanish-speaking grandmother was his participating caregiver, his bilingual mother completed screening and preferred English assessments. The CARS-2 and the GARS-3 were used to describe ASD characteristics. The CARS-2 is a rating scale that provides a reliable and valid measure of the presence and relative severity of ASD characteristics (Reszka et al., 2014). Because the CARS-2 is not available in Spanish, the Spanish GARS-3 (which has demonstrated reliability and validity across measures; Schmidt et al., 2023) was used with Zandra’s mother. Child communication skills were assessed via the Communication Matrix (Rowland, 2011), and the Vineland Adaptive Behavior Scales 3rd Edition (VABS-III; Sparrow et al., 2016). The Communication Matrix is available in English and Spanish and is a broadly used expressive communication assessment (Rowland, 2011). The communication sections of the VABS-III were used to further document language skills. The English VABS-III interview form was completed by the first author with Gustavo’s mother and the Spanish parent/caregiver form was used with Zandra’s mother. Children’s preferences were assessed using English and Spanish versions of the Reinforcer Assessment for Individuals with Severe Disabilities (RAISD; Fisher et al., 1996, 2021).

### 2.2. Researchers 

The lead instructor (BCBA-D, an associate professor in SLP department, and final senior author) provided instruction and coaching to student clinicians. The lead instructor (a non-Hispanic white female) completed 4 years of high-school Spanish, and two intermediate-level college Spanish courses (including one abroad in Argentina) but did not use Spanish regularly in conversation at the time of the study. The instructor’s receptive Spanish understanding exceeded her expressive use, and she did not have the conversational proficiency to coach in Spanish. The first author was a Hispanic master’s level SLP student who was bilingual Spanish speaker with an advanced level of proficiency, acquiring her heritage language later in life. The second author was a neurodivergent SLP/PhD student from a Hispanic background with minimal Spanish skills. The third author was a bilingual, white, non-Hispanic PhD student/SLP who uses Spanish daily with her Spanish-speaking bilingual husband, mother-in-law, and children. The fourth author was a Latino, heritage speaker of Spanish, assistant professor of speech and hearing sciences, and licensed SLP. An undergraduate bilingual, heritage speaker of Spanish, provided accuracy checks of materials and interview transcripts.

### 2.3. Setting, Materials, and Cultural Adaptations

All sessions took place in child-friendly research rooms in a university clinic. Each room contained a small table with chairs and a carpeted play area, as well as visual supports and play/activity materials described below. Amazon gift cards ($10/session) were used as incentives for caregiver participation in coaching, and student clinicians and caregivers received a $25 gift card for interviews. Visual supports adapted from Gevarter et al. (2021) and Gallegos et al. (2024) were translated by the first author (checked by heritage Spanish speakers) and are available in Supplementary Materials. Supports included English and Spanish versions of (a) instructional visual aids that outlined NDBI techniques, (b) activity planners used to collaboratively joint plan sessions with clinicians and families, and (c) coaching checklists. Video models of NDBI and coaching techniques (Gevarter et al., 2021) were used during student group instruction.

Cultural adaptations utilizing the EVF (Bernal et al., 1995) were incorporated throughout the creation of coaching materials and procedures. More specifically, as part of the research planning process, authors 1 and 5 regularly met with author 4 (who has expertise in culturally responsive interventions) to discuss possible adaptations that were rooted in prior research and to receive support and recommendations regarding translation of materials. Some examples of research-derived considerations included not requiring that caregivers: (a) use lengthy wait times or (b) provide extensive play narration (DuBay et al., 2018; Gallegos et al., 2024; Gevarter et al., 2021). Rationale building and discussion during joint planning was also used to address potential concerns with using environmental arrangements, following a child’s lead, and matching language to a child’s skills (Cycyk et al., 2020; DuBay et al., 2018; Rollins et al., 2024). Additionally, during active data collection authors 1, 3, and 5 (who were involved in direct implementation) met on a weekly basis to discuss issues and concerns and make additional adaptations as needed. Table 3 provides examples of both pre-study implementation adaptations as well as individualized adaptations made during the study.

Using parent-provided information from the RAISD (Fisher et al., 1996), researchers selected child specific sets of play materials (one set for student-clinician-led sessions, and another for caregiver-led sessions) that included individualized, developmentally and culturally appropriate preferred items and activities. Access to larger items such as a water table remained consistent across all sessions. Preferred items were placed in clinic rooms inside a bag at the beginning of each session. Gustavo’s items included cars/car ramp, cause-and-effect toys (e.g., balloon toy), a water table, and balls. Initially, Zandra’s items included a water table, music toys, playdough, and music books. While these items were used throughout Zandra’s sessions, during baseline Fernanda mentioned that she was not accustomed to interacting with Zandra during play, and more often engaged with Zandra during mealtimes. Thus, food items (e.g., gummies, pizza) were added as an adaptation for caregiver-led sessions. An additional adaptation for Zandra included adding an iPad with the Proloquo2go application (by Assistiveware) during coaching. Individual pages corresponding to Zandra’s preferred items were created. Each page included one speech output button (photograph of a preferred item/activity), measuring 8 by 5 inches (see Supplementary Materials). Spanish speech output used a digitized child Spanish speaker voice. Authors 1 and 5 worked with Author 3 (a bilingual SLP specializing in AAC) to make decisions regarding the display and vocabulary. These decisions were further refined during joint planning with the student-clinician and caregiver. Vocabulary items selected for the AAC device corresponded to Zandra’s play/meal activities/items and communication temptations.

### 2.4. Research Design

**Figure 1:**
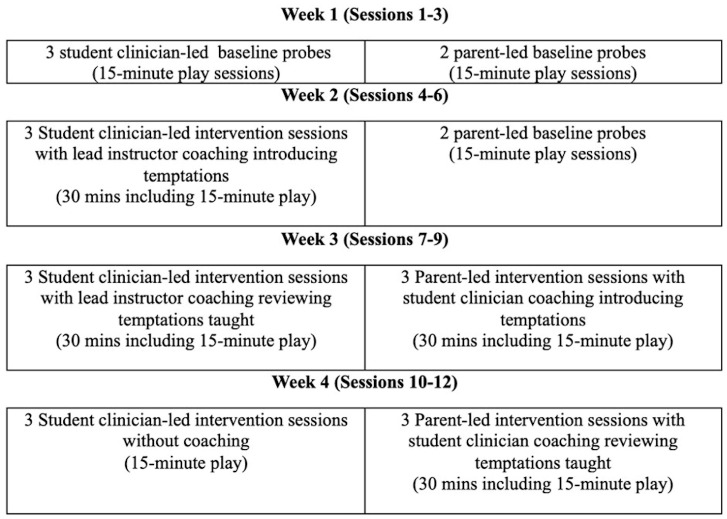
Research Design Timeline.

This multiple methods study included a multiple probe across (adult) participants single case experimental design (Gast et al., 2014) and semi-structured qualitative interviews. Research sessions took place during a four-week summer clinic. Three days a week, children and student clinicians participated in group sessions formatted similarly to preschool. Caregiver-led sessions took place prior to the start of daily group sessions. Daily student-led sessions occurred on a pull-out basis at least one hour after caregiver-led sessions. During week one, baseline probes occurred over three student-led and two caregiver-led sessions. During week two, caregivers remained in baseline (i.e., two additional probes), while the lead instructor provided student group instruction in NDBI and initiated coaching during student-led sessions. During week three, the lead instructor continued to coach students during student-led sessions (fading support as needed) and provided student group instruction in coaching. If students increased use of targeted NDBI techniques for at least two student-led sessions during week two, students then initiated caregiver coaching sessions in Spanish (after group instruction in coaching) during week three. In week four, student-led sessions continued without the lead instructor, and student clinicians continued to coach caregivers independently. Figure 1 provides an illustration of the intended research timeline. Due to illness, Triad 1 only completed six student-led intervention sessions and five caregiver-led sessions. 

After experimental sessions concluded, the first author completed social validity semi-structured interviews with each adult participant. Semi-structured interviews have been used to effectively gather the perspectives of Hispanic parents of autistic children (Hampton et al., 2017; Rollins et al., 2024).

### 2.5. Dependent Variables 

The primary dependent variables were the student clinician and caregiver’s rate of targeted NDBI elicitation and response techniques per minute. Elicitation techniques included (a) using a targeted communication temptation, (b) waiting at least 3 s for a communicative response, and (c) prompting a response if no communication occurred. Individualized prompts were selected after baseline and could include models, gestures, or physical cues. Verbal cues such as asking questions or providing directives (e.g., *What do you want?*) were not considered prompts but rather natural cues. Response techniques included (a) reinforcing communication with natural consequences such as completing actions or giving items requested, (b) pairing reinforcement with a contextually related speech model describing the child’s communicative turn, and (c) allowing the child to interact with items/activities. Contextually related models could be of any length (e.g., *help* or *you need help*), but did not include statements that only involved praise (e.g., *good job!*) or conversational responses such as *thank you.* There was no criterion for utterance length or grammatical completeness as prior research suggested this was challenging for English-speaking parents to acquire in a short period (Gallegos et al., 2024). For interaction, the adult provided time for the child to interact or play prior to initiating another temptation.

### 2.6. Data Collection 

#### 2.6.1. Coding of Dependent Variables and Interobserver Agreement (IOA) 

All sessions were videotaped, and coders used a standardized data collection sheet to document components of dependent variables. These included whether the adult (a) used a targeted temptation, (b) waited 3 s or more before prompting, (c) provided a prompt if needed, (d) reinforced the child’s communication, (e) provided a contextually relevant speech model, and (f) allowed the child to interact with items/activities. If steps a–c were correctly implemented, the coder recorded that elicitation techniques were used correctly. Similarly, if steps d–f were correctly implemented, the coder recorded that response techniques were used correctly. The first and third authors each served as the primary coder for one triad, and the independent observer for the other. For each participant, 33% of sessions were randomly selected for IOA. The number of agreements was divided by the total number of disagreements plus agreements. Average IOA was: 97% and 90% for Triad 1 student-led and caregiver-led sessions, respectively; 93% and 88% for Triad 2 student-led and caregiver-led sessions, respectively.

#### 2.6.2. Semi-Structured Interviews

Interviews were conducted in person or via Zoom (based on participant preference) and audio recorded/transcribed. Interviews included a set of predetermined questions to guide discussion. Follow-up questions were used to clarify and expand upon responses (DiCicco & Crabtree, 2006). Author 1 developed draft questions and potential follow-up questions which were then edited and refined based upon feedback from authors 4 and 5. Questions were designed to gather information regarding (a) a priori themes of program effectiveness and areas for improvement and, (b) participants’ perceptions of child outcomes. These questions align with social validity constructs regarding the social appropriateness of procedures, and the social importance of treatment outcomes (Ferguson et al., 2018). Both student interviews and Martha’s interview lasted 20 min. As Fernanda expanded on answers, her interview was an hour.

### 2.7. Procedures for Multiple Probe Design 

#### 2.7.1. Baseline and Target Selection 

During baseline, adult participants were instructed to interact/play with the child as they normally would. Once the child showed interest in and/or engaged with an item or activity, a 15 min observation began. Students primarily used Spanish with some English words and/or phrases within student-led sessions. The lead instructor was present during student sessions but did not provide coaching or feedback. All caregiver baseline probes occurred in Spanish. Students were present during caregiver-led sessions but did not provide coaching or feedback.

Researchers reviewed baseline sessions and selected target communication temptations for each adult participant from a list adapted from Gallegos et al. (2024). Targets were chosen if they were used fewer than three times across two or more sessions and were appropriate for the child’s interests/activities. Infrequently used temptations were selected to introduce new skills to clinicians and caregivers and increase the variety of child communicative forms/functions. Communication Matrix data and baseline observations were used to create a list of multimodal communication forms a child might use in response to temptations. Initially, due to the short duration of the study there was no intention of introducing an entirely new communication system (e.g., aided AAC) to either child. An adaptation was made to introduce the Proloquo2go AAC application for Zandra during coaching. Zandra used limited gestures during baseline, was observed during group clinic times to show an aversion to physical prompting of signs or gestures, and her mother reported that Zandra became easily frustrated whenever Fernanda tried to prompt natural speech. Following baseline, the researchers had a conversation with Fernanda about the benefits of introducing aided AAC. Fernanda expressed a strong preference for using an AAC application over signs. During joint planning, it was also decided to continue to accept Zandra’s use of persistent vocalizations (e.g., *ah-ah-ah*) and pre-existing gestures to reduce frustration. As there was no report or observation of aversion to prompting unaided systems for Gustavo (who was reported to have recently started using some signs and words just prior to the study), unaided forms were selected for his targets. During coaching, the lead instructor discussed, clarified and modified child response options with student clinicians, who did the same with caregivers.

Table 4 outlines targeted temptations, along with example child responses.

#### 2.7.2. Group Instruction and Coaching of Students

At the start of week two, the lead instructor provided students’ instruction in NDBI during a university course. Prior to the in-person course, students were required to complete an online module on Naturalistic Intervention (Amsbary & AFIRM Team, 2025a) and read chapters on goal development and targeting communication skills (Minjarez et al., 2020a) in the NDBI textbook by Bruinsma et al. (2020). During the course lecture, although specific manualized NDBI models were discussed, given the short-term nature of this study and students’ lack of access to more extensive and paid trainings, the lead instructor focused on reviewing NDBI techniques that are common across various manualized programs (Bruinsma et al., 2020). For the last hour of the course, a behavioral skills training (BST) model (Miltenberger, 2011) was used to describe, model, rehearse, and provide feedback on the specific NDBI communication techniques students would be required to use during this study. These techniques were adapted from prior research (Gallegos et al., 2024; Gevarter et al., 2021) as well from the course reading/training materials (Amsbary & AFIRM Team, 2025a; Minjarez et al., 2020a). Specifically, elicitation techniques taught included (a) creating a temptation via environmental arrangements (b) waiting for a child to communicate and (c) prompting communication if none occurred. Response techniques taught included (a) using a natural consequence to reinforce prompted or unprompted communication, (b) providing a contextually relevant spoken language model mapped to the child’s communication, and (c) providing time for the child to interact with materials prior to initiating another temptation. The interaction technique was added based upon findings from Gallegos et al. (2024) which indicated that some participants over-prioritized creating temptations leading to less focus on engaging in play with the child, thus limiting the naturalness of the intervention. During BST the instructional visual aid, activity planner, in situ models, and video models were introduced. Roleplay and feedback occurred in small groups. Although the focus of this study was specifically on NDBI communication techniques, BST did embed instruction regarding (a) how to increase motivation for temptations by varying play routines and following a child’s lead and, (b) different ways to engage in play with a child during reinforcement and interaction. As part of the associated course, during the last week of the study, students did receive further instruction in NDBI techniques for fostering peer inclusion and improving social skills and play (Bruinsma et al., 2020).

After group instruction, the lead instructor began coaching students. Coaching sessions lasted approximately 30 min, including the 15 min play interaction. The coaching checklist was used to maintain fidelity. At the start of first coaching session, the instructor reviewed the instructional visual aid (in English and Spanish) and collaboratively filled out the activity planner (in English) with the student while modeling techniques with the child. First, the coach introduced and modeled one target temptation, and then collaboratively brainstormed activities aligned with the temptation. The instructor then provided a wait time rationale and discussed when to wait longer than three seconds. Next, the instructor and student identified child communication responses to accept and prompt. They selected two prompts (e.g., graduated physical guidance, models) appropriate for the child, and the instructor modeled these prompts along with natural reinforcement techniques. Lastly, the instructor and student brainstormed examples of contextually relevant Spanish spoken labels, and examples of how the child could interact with items. After joint planning, student clinicians led the 15 min play sessions with the child while primarily speaking Spanish. During play, the instructor provided verbal cues/feedback to the students, assisted with materials, and helped redirect children when needed. After play, the instructor gave students a mix of constructive and positive feedback on at least three NDBI techniques, and students were given time to ask questions. Coaching sessions two-three followed the same format, introducing one new temptation a day. During sessions four-six, no new temptations were introduced, and the visual aid and activity planner were available. During the first 5–10 min, students could ask questions and skills were reviewed as needed. Students again ran the 15 min play sessions while the instructor assisted or cued as needed. After play, the instructor provided feedback and time for questions. During the final three sessions (if child participant attended all sessions) the student ran sessions independently (instructor not present). Notably as procedures were designed to be individualized to a variety of multimodal communication forms, changes to the coaching process were not required when the AAC device was introduced for Zandra. For instance, similar to the process for unaided forms, during joint planning the coach and student clinician discussed and selected prompt options (e.g., modeling) that would be appropriate for aided AAC.

#### 2.7.3. Student Group Instruction in Coaching and Student-Led Caregiver Coaching

At the start of week three, student clinicians were first required to complete an online module on parent-mediated intervention (Amsbary & AFIRM Team, 2025b) and read the course textbook chapter on parent coaching (Minjarez et al., 2020b). At the start of class, students first discussed a variety of more general strategies for working with families. During the last hour of the course BST was used to introduce the coaching steps that the lead instructor followed while coaching students. These steps were derived from prior research (Gallegos et al., 2024; Gevarter et al., 2021) as well as from course materials (Amsbary & AFIRM Team, 2025b; Minjarez et al., 2020b). Specifically, these included (a) joint planning with the activity planner (first three coaching sessions) or pre-session check-ins (last three coaching sessions); (b) describing and modeling techniques with the aid of visual supports (first three coaching sessions), or providing visual aids and reviewing/modeling as needed (second three coaching sessions); (c) setting up and observing the 15 min caregiver/child play and providing verbal cues/feedback or assistance as needed and (e) providing post-session feedback addressing at least three of the communication techniques. The instructor described and modeled these coaching steps and provided students with coaching checklists in English and Spanish. Students worked together to translate descriptions of temptations and other individualized techniques (e.g., specific prompts) into Spanish terms they would use with caregivers. The students practiced implementing coaching steps via role play and the lead instructor provided feedback. It should be noted that Gustavo’s clinician (Nadia) mistakenly wrote down giving choices as one of Martha’s targeted temptations, although the researchers had selected inadequate portions. As this was not discovered until after Nadia began coaching Martha, giving choices remained a target. 

After the group instruction, student clinicians began in-session caregiver coaching. Procedures were identical to lead-instructor coaching except the student clinicians coached caregivers in Spanish and all materials provided to caregivers were in Spanish.

### 2.8. Treatment Fidelity

To ensure group instruction fidelity, the lead instructor used a checklist outlining BST steps while conducting group sessions. The first author used the same checklists to confirm 100% fidelity for both group sessions. Coaching fidelity checks were conducted using the videos coded for IOA (33% of coaching sessions). The first and third authors used checklists to mark steps used during coaching by the lead instructor and students. Average lead instructor coaching fidelity was 97% for Triad 1 and 100% for Triad 2. Average coaching fidelity for Nadia (Triad 1) was 85%, and average fidelity for Raquel (Triad 2) was 97%. A fidelity check-in with the instructor and students occurred after early checks indicated that students were sometimes implementing NDBI steps themselves instead of allowing caregivers to use techniques. The check-in consisted of a 10 min discussion clarifying this issue and addressing questions. 

### 2.9. Data Analysis 

#### 2.9.1. Experimental Design Analysis

The two primary dependent variables were graphed across baseline and coaching phases. Baseline and intervention differences in trend, level, variability, and immediacy of effect were examined via visual analysis (Kratochwill et al., 2013). Tau-U, a non-overlap effect size measure, was also used as it is compatible with visual analysis, controls for positive baseline trends, and accounts for change in level and trend between phases (Brossart et al., 2014; Rakap, 2015). Tau-U was calculated using a web software package (Vannest et al., 2016), applying a 90% confidence interval. Effect sizes were categorized based on parameters defined by Vannest and Ninci (2015), where 0.20 and below suggested a small effect, 0.20–0.60 indicated a moderate effect, 0.60–0.80 denoted a large effect, and 0.80 and above indicated a very large effect.

#### 2.9.2. Social Validity Interview Analysis 

Interview audio recordings were transcribed by an online service (Scribie) that provides transcription for both English and Spanish recordings. Spanish-language interviews were not translated to preserve the original meaning and integrity of the participants’ responses. Instead, researchers collaborated with a native Spanish speaker to confirm accurate interpretation throughout analysis. The first and second authors utilized a reflexive thematic analysis, as outlined by Braun and Clarke (2006). Throughout the coding process, first and second authors independently conducted open coding for each participant group (student clinicians followed by caregivers). They then met to discuss and refine codes, reaching consensus through iterative, collaborative dialog. For the caregiver interviews, authors worked closely with the heritage Spanish-speaking undergraduate student during open coding to ensure the preservation of original meaning. First and second authors then organized codes into a priori categories (effective program elements, areas for improvement), which were then developed into emergent themes and sub-themes. Finally, first, second and final authors met to review and refine themes within and across participant groups, reaching consensus on the final thematic structure. The first author maintained a journal and memos to explore positionally and reflexivity.

## 3. Results

Findings across triads demonstrated functional relationships between the culturally adapted NDBI cascading coaching model and dependent variables. Experimental results are described per triad. Social validity findings are summarized across participants. 

### 3.1. Triad 1 Experimental Results

**Figure 2:**
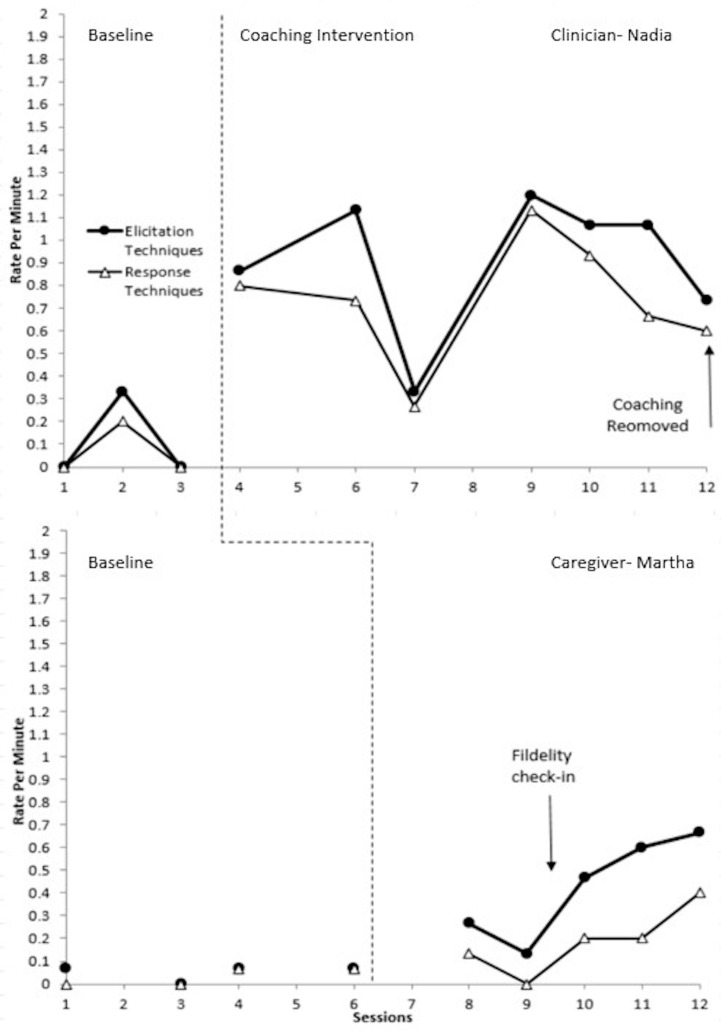
Triad 1 student clinician and caregiver rate of targeted elicitation and response techniques per minute.

The data for Triad 1 is displayed in Figure 2. For elicitation techniques, very large effect sizes of 1 (CI = 0.69–1.31) for the student clinician, Nadia, and 1 (CI = 0.67–1.33) for the caregiver, Martha, were observed. For response techniques, Nadia had a very large effect size of 1 (CI = 0.69–1.31) and Martha had a large effect size of 0.66 (CI = 0.86–1.14).

Nadia exhibited some variability in technique use during baseline, as she used some targeted temptations during the second session but not in the other two baseline sessions. Following the introduction of coaching, Nadia showed an immediate increase in her use of both targeted elicitation and response techniques. Although all data points for both dependent variables remained above baseline throughout coaching, there was variability and an overall neutral trend. A decrease in the use of elicitation and response techniques during the third session occurred due to the researchers cutting the play session short by six minutes after the child withdrew assent to participate (rejecting materials, crying, and trying to leave the room). The caregiver reported the next day that the child was sick, and he missed the following two sessions. Around this time, he also began showing difficulty with leaving his grandmother. His session times were adjusted to provide him with more time to transition. Afterwards, there was an increase in Nadia’s use of techniques, which then remained relatively stable until a slight decrease in the last session when coaching was removed. Overall, Nadia’s rate of response techniques was lower than her elicitation technique use. This was primarily due to responding with *gracias* when the child would hand over an object to request assistance rather than using a contextually relevant model of the child’s turn. There was also more of a decline in her response technique rate during the last several sessions. However, even when instructor coaching was removed, elicitation and response technique use remained well above baseline levels. 

Martha maintained generally low stable baselines, with only minor variability. After student-led coaching, Martha showed a slight but immediate increase in level in her rate of both targeted elicitation and response techniques. Overall, there was a gradual increasing trend across her use of elicitation techniques and response techniques. During the second coaching session, her rate of both dependent variables decreased due to the student clinician implementing some techniques herself. Following the fidelity check-in, there was an immediate increase in Martha’s rate of technique use continuing gradual upward trends. Overall, Martha used fewer response techniques than elicitation. This was primarily due to responding to the child’s communicative turn by using words of praise or affirmation (e.g., *bravo*, *gracias*, *OK*) or nodding her head instead of responding with a speech model.

### 3.2. Triad 2 Experimental Results

**Figure 3:**
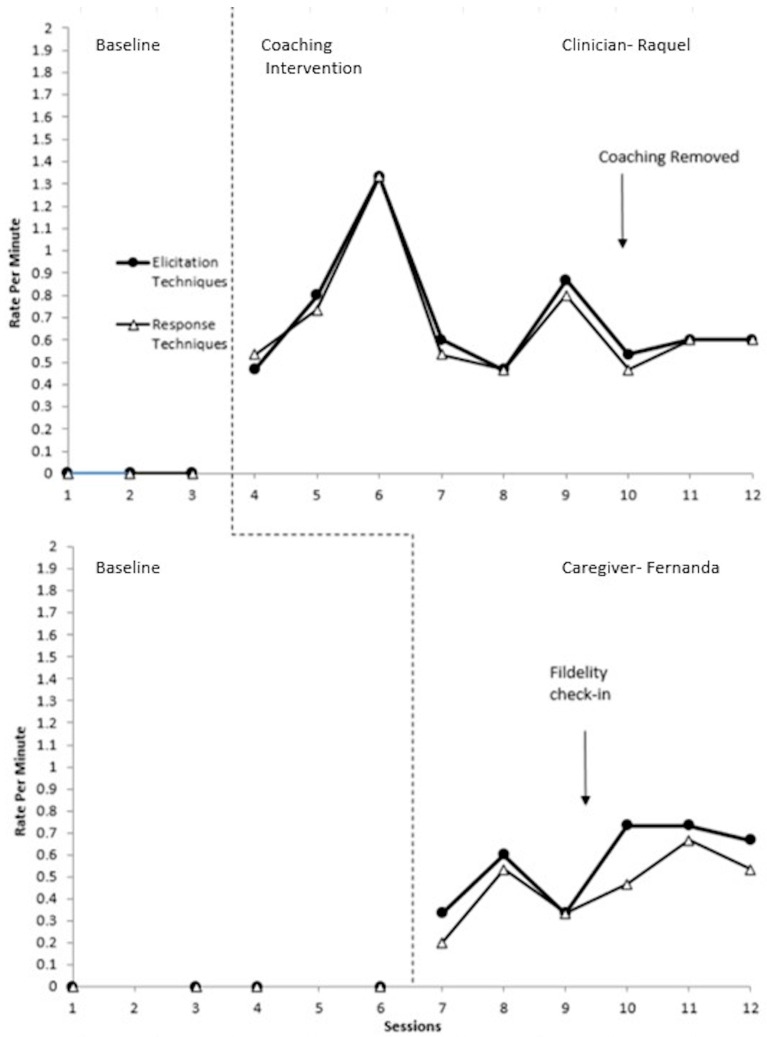
Triad 2 student clinician and caregiver use of targeted elicitation and response techniques.

Data for Triad 2 is displayed in Figure 3. For elicitation techniques, very large effect sizes of 1 (CI = 0.66–1.34) for the student clinician, Raquel, and 1 (CI = 0.64–1.36) for the caregiver, Fernanda, were observed. Both also had very large effect sizes for response techniques, which were 1 (CI = 0.66–1.34) for Raquel and 1 (CI = 0.64–1.36) for Fernanda.

Raquel maintained a stable baseline of zero targeted elicitation and response techniques per minute. After coaching began, there was an immediate increase in her rate of elicitation and response techniques. Data trends were relatively neutral, but during the third session, Raquel had a particularly high rate of technique use due to the child independently initiating communication more frequently (e.g., immediately reaching for another toy right after a prior communicative act was reinforced). Raquel consistently and equally used elicitation and response techniques across all sessions including when coaching was removed.

Fernanda maintained a stable baseline of zero targeted elicitation and response techniques per minute. Once coaching was introduced, there was a gradual increasing trend in both elicitation and response techniques, with some variability across both. There was a decrease in her use of elicitation and response techniques during the third coaching session, as the student clinician implemented some techniques herself. Following the fidelity check-in, Fernanda’s rate of technique use continued to show gradual upward trends for the remainder of sessions. Overall, Fernanda used slightly fewer response techniques than elicitation. She sometimes did not provide a speech model or responded using only affirmative words such as *eso* (*that’s it*).

### 3.3. Social Validity Results

Findings from interviews were divided into two a priori categories: caregiver and student clinician perspectives on (a) areas for improvement, (b) effective program elements. Emergent themes reflected improvement needs or effective changes regarding participants’ *learning*, *perspective*, *behavior*, and *relationships*. Within each intersection of the a priori categories and emergent themes, subthemes were also coded. Table 5 provides an overview of the themes and subthemes which are further described under the larger categories below. For caregiver quotes, researchers provided the original quote in Spanish and an English translation.

#### 3.3.1. Area for Improvement-Learning

Both student clinicians and caregivers recommended extending the duration of the program. Raquel noted, *More trainings would be helpful to mom, and to me as well*. She also suggested extending sessions to 45 min or an hour. Additionally, Nadia envisioned *A sit-down last session with [Martha] where I’m like, Okay, do you have any questions*? She felt this would have been beneficial since, *even up to the last point, we were just practicing or giving her [Martha] feedback*. Students also highlighted additional translation needs. Nadia expressed challenges with translating additional terms taught in class, and both students sought out additional resources to adapt their explanations for caregivers. Nadia described her approach as reflecting on *Does that sound appropriate or does that sound like something that she [Martha] would understand*? Similarly, Raquel shared *I would just look it up, and then I would come up with my own word to teach it to mom, like something that she could understand.*

#### 3.3.2. Area for Improvement-Perspectives

Adult participants identified a need to address feelings associated with learning new skills in a research environment. For instance, Martha stated *Al principio pensaba que iba a ser algo muy difícil… no iba a poder hacer nada* (*At first, I thought it was going to be something very difficult…. that I was not going to be able to do anything*). Raquel echoed, *At first, I was a little nervous that I was going to mess up the study… and then I was also nervous because I was getting videotaped.* Nadia added, *It was overwhelming at first because I didn’t know what to expect or what to do or with the research* and *I just hope that I did a good job in telling her [Martha], getting the main points across so that he [the child] is achieving his goals*.

#### 3.3.3. Effectiveness-Learning

Participants agreed that the program led to a positive learning experience. Nadia shared, *I really, really enjoyed it [the program]* while Raquel reflected, *It was a wonderful experience, and I really liked it.* Caregivers also noted that the child’s enjoyment enhanced their experience. For instance, Maria expressed, *yo pienso que también lo disfrutó él y me emocionaba ver* (*I think that he also enjoyed it and it thrilled me to see*).

Additionally, participants indicated that the program allowed for accessible learning. As Fernanda explained, *los horarios son muy buenos* (*the hours are very good*) *and los servicios de ustedes… llegaban a todas las partes del estado, no tenían un límite* (*Your services, they included all parts of the state, they weren’t limited*). Martha (who participated because Gustavo’s parents had to work) also explained, *Cuando uno trabaja no es lo mismo y claro que quién mejor que sus papás para compartir esto con él, ¿verdad? Pero no, no se pudo* (*When someone works it is not the same and of course, who would be better than his parents to share this with him, right? But no, they couldn’t*). 

Caregivers and students also expressed satisfaction with the dedicated time spent working with families. Fernanda shared, *El éxito del programa es el tiempo que pasan con los niños* (*The success of the program is the time spent with the children*). Raquel reflected that coaching, in comparison to other models, provided more learning. She explained, *There’s a lot of benefits to the parent training because they actually learn what to do instead of, oh, you bring the child back into the therapy room, but parents don’t actually know what you’re doing, and they can’t actually do that in their homes.* In addition, Fernanda shared the difference she noticed between the time her student clinician was able to provide compared to Zandra’s school SLP. She expressed, *Fue muy poco el tiempo, pero los avances fueron muchos, muchos, muchos… [the school SLP] está saturada en sus consultas, cómo tiene una lista de espera de meses. Y entiendo, ella quisiera darle servicios a todos, pero no puede. Ustedes todavía no tienen eso. Ustedes son estudiantes y están concentrados en aprender y en enseñar lo que saben. Entonces, creo que eso es muy bueno y ha sido muy bueno para nosotros.* (*It was a very short amount of time, but the progress was so much… …the [School SLP] is swamped with appointments and has a wait list of months. And I understand, she would love to give everyone services, but she can’t. You all don’t have that yet. You’re students and you’re focused on learning and on teaching what you know. And I think that’s a good thing and it’s been very good for us*).

Proactive, individualized, and culturally responsive support for both adults and children was also key to effective learning. For instance, despite the need for additional translation support needed, Raquel noted, *The resources that you gave us that you translated from English to Spanish, that really helped.* Fernanda similarly emphasized, *Es increíble cómo todos están siempre tratando de comunicar con nosotros en nuestro idioma* (*It’s incredible how everyone was always trying to communicate with us in our own language*). This helped her fully grasp the material: *Entiendo todo lo que ustedes decían, toda la información, a todas sus preguntas en Español* (*I understood everything that you all said, all the information, all of your questions in Spanish*). Speaking in Spanish also enhanced Nadia’s learning experience. She explained, *It’s just easier for me to express myself in Spanish and understand them* [Spanish speaking families] and *I felt more comfortable with the Spanish-speaking families as well because I just feel like I relate to them a little bit more.* Regarding individualized child support, Raquel shared that having the flexibility to choose which child communication responses to accept or prompt *really helped a lot.* Another effective personalized child support was the introduction of aided AAC for Zandra. Fernanda reflected on its impact, stating, *El iPad y el programa que tiene el iPad le ha ayudado mucho a Zandra a que no se frustre tanto. Ella sabe que tiene un medio de comunicación que es como si fuera su voz. Ella ha aprendido a hacerlo y a comunicarse a través de eso y creo que ella tiene muchas posibilidades de seguir aprendiendo a través del iPad a comunicarse más* (*The iPad and the program on it has helped Zandra so much so that she no longer gets as frustrated. She knows that she has a means of communication that is like her own voice. She has learned how to use it and how to communicate through it and I believe that she has many possibilities to continue learning through the iPad and to communicate more*).

Lastly, participants expressed the benefits of the cascading model. As Raquel described, *Towards the end, I started getting it more and more, and then I was able to teach mom how to do that as well and I was really excited that I learned because now Zandra knows how to communicate more.* Martha also indicated that she generalized the cascading model to other non-participating family members, sharing that she regularly updated Gustavo’s parents: *Cada día llegaba y les platicaba [a sus papás]. Le decía [a su mamá]: ‘Mira, este hoy pasó esto, me dijeron esto, se habló de esto, o jugamos así* (*Every day I would arrive, and I would chat with them [his parents]. I would tell her [his mom] ‘look, today this happened, they told me this, we talked about this, or we played like this,’ I would always tell them about it*). 

#### 3.3.4. Effectiveness-Perspectives

Interviews indicated that the program shifted participants’ perspectives on NDBI. Fernanda demonstrated her newfound understanding of the importance of natural interactions and speech models: *Necesitamos que nos involucremos con ella, que hablemos con ella… que es parte de su aprendizaje, de su desarrollo, el hecho de que estemos jugando, porque ella es su forma de que va a aprender, que estemos mencionando palabras… Y ella va a ir identificando lo que nosotros decimos a través del juego con ella, pero de una forma cercana y por un lado de ella* (*We need to be involved with her, talk with her, this is part of her learning, her development, all while we are playing, because this is her way that she is going to learn, while we are mentioning words…and she’s going to identify what we are saying through playing with her, but in a close way, alongside her*). She then reflected on the value of wait time, stating, *Algo que aprendí, que tengo que esperar a que ella se comunique. Tengo que esperar, tengo que darle su tiempo y no frustrarla* (*Something that I learned, I have to wait for her to communicate. I need to wait, I have to give her time and not frustrate her*). Raquel also reinforced key benefits of NDBI stating, *It was a nice learning experience because I have never really participated in a naturalistic environment, but it was also structured.*

#### 3.3.5. Effectiveness-Behaviors

All adult participants reported improvements in the children’s communication. Nadia reflected on Gustavo’s progress, saying *At the beginning, he was really, really, quiet…. He relied a lot on his grandma and kind of his way of communicating was more guiding and more pulling one to what he wants…. towards the end of the study, it was a lot more of using those gestures… those signs that we had taught him…he was able to express himself in different ways… So I saw that difference, and also the grandma saw that difference as well, which was great to see.* Martha further confirmed this explaining, *En cada cosa, en cada vez que veníamos, yo notaba algo en él, aunque fuera una palabra, aunque fuera o así* (*In everything, every time we came, I noted something in him, whether it was a word or a sign*)*. She also shared ya tenemos mejor comunicación de muchas maneras* (*We now have better communication in many ways*). Regarding Zandra’s communication, Raquel reflected that, *At the beginning, I feel like she would get mad really easily, and it was hard for her to regulate, and she would start crying and throw herself to the floor.* Raquel then compared this to the end of the program sharing, *Now… she knows that if you use your words or you use the iPad to communicate, she’s going to get what she wants*. Fernanda confirmed this change in Zandra’s communication, sharing, *a través del programa, Zandra ha podido expresar más, no solamente no sólo deseos, sino emociones* (*Through the program, Zandra has been able to express herself more, not only her wants, but also her emotions*). She also described long term benefits: *yo sé que ella aprendió muchísimo y son aprendizajes que ella va a ser un inicio para el resto de su desarrollo* (*I know that she learned so much and that this learning will be the beginning of the rest of her development*).

Participants also reflected on their own/the caregiver’s increased use of NDBI skills. Raquel reflected *[It was] really nice to learn how to, [and] what to respond to Zandra, instead of just giving her what she wants right away.* She also noted changes in Fernanda’s implementation of NDBI, saying, *From the first parent training, to this one, she changed a lot. She stopped giving her directions. She would listen to my feedback, and she would change.* Fernanda further reflected on how her use of NDBI skills contributed to Zandra’s improvements. She explained, *Ella también entendió me van a esperar, entonces no tengo por qué enojarme. Mamá va a esperar que yo le exprese lo que necesito’. Entonces, ella ahora pide las cosas, pero… de forma más tranquila, sin estresarse tanto* (*She also understands they are going to wait for me, so I don’t need to get angry. My mom is going to wait for me to express what I need to. So now she requests things, but …in a more calm manner, without stressing herself so much*).

#### 3.3.6. Effectiveness-Relationships

Both caregivers reported positive changes in their relationships with child participants. Martha reflected, *Me gustó mucho porque todo, para mí, todo lo que aprendí, lo que escuché, para mí todo era bueno por el niño, para poder tener mejor convivencia con él y saber cómo compartir las cosas y todo* (*I liked it a lot because everything, for me, everything that I learned, that I heard, everything was good for [my grandson’s] sake to be able to live better together and to know how to share things and everything*). Similarly, Fernanda expressed, *Aprendí también que puedo conectar con ella, convivir con ella, interactuar con ella a su forma* (I *also learned how to connect with her, live better with her, interact with her in her way*). She further explained that this shift brought her entire family closer together, sharing: *Normalmente jugaba ella aparte, yo aparte, su hermana aparte, todos aparte. Y ahora sabemos que podemos estar juntas y que ella nos acepta* (*Normally she played separate, me separate, her sister separate, everyone separate. But now we know that we can be together and that she accepts us*).

## 4. Discussion

Findings demonstrated that a brief cascading coaching model, led by a BCBA-D with limited Spanish conversational skills, resulted in an increase in the use of NDBI techniques amongst bilingual graduate SLP student clinicians and monolingual Spanish-speaking caregivers of autistic children. All participants had very large effect sizes for the use of elicitation techniques, and three participants demonstrated a very large effect size for the use of response techniques (one demonstrated a large effect size). Findings are congruent with similar studies teaching clinicians and caregivers to use NDBI via cascading coaching (e.g., Gallegos et al., 2024; Gevarter et al., 2021). Additionally, both experimental and qualitative findings provide further support that NDBIs can be culturally adapted to meet the needs of Spanish-speaking families (Douglas et al., 2024; Lopez, 2024; Meadan et al., 2020; Pickard et al., 2024). Such findings are particularly important for improving equitable access to evidence-based early autism intervention services for Latino families (Guerrero & Sobotka, 2022; Magaña et al., 2013; Zuckerman et al., 2017).

In terms of experimental findings, while all participants had an increase in their use of NDBI techniques, visual analysis revealed variability among participants consistent with similar studies (Gallegos et al., 2024; Gevarter et al., 2021). Prior research has shown that eliciting between one and two communication turns per minute is natural and appropriate for early language learners, while also being effective in increasing target behaviors (Coogle et al., 2015; Hamberger et al., 2022). Although participants did not always meet this rate, overall rates of communication opportunities were likely higher when considering non-targeted (e.g., previously mastered) temptations. Additionally, variability is expected when implementing NDBI with young children as adults must follow the child’s lead and respond to varying interests and engagement (Gallegos et al., 2024). Variability across participants may also be reflective of differences in communication partners’ relationships to the child (Gallegos et al., 2024). For instance, in comparison to Martha, Fernanda (child’s mother) tended to more frequently prompt Zandra to use her higher-level communication form (aided AAC device), while Martha (child’s grandmother) more often accepted lower-responses (e.g., gestures) rather than prompted Gustavo to use higher level forms (signs, words).

Qualitative data also highlighted key aspects that contributed to areas for improvement the program’s effectiveness. Adult participants expressed a desire for an increased study duration, additional translation support, and greater acknowledgment of feelings involved with learning new skills in a research setting. Participants reported an overall positive learning experience and noted that the program’s accessibility, dedicated time spent with families, child and adult culturally relevant and individualized support, and the cascading model all contributed to learning. Participants also gained new perspectives on the benefits of NDBI and reported improvements in children’s communication as well as their own NDBI implementation. Finally, caregivers shared that the relationships with their children improved.

Both experimental and qualitative results support prior qualitative findings regarding varying levels of difficulty caregivers experience with specific NDBI skills (DuBay et al., 2018; Rollins et al., 2024). For instance, while Fernanda initially had difficulty implementing wait time and was more directive, Martha demonstrated a more child-led approach and used wait time naturally. The fact that Fernanda expressed positive views about wait time and following Zandra’s lead during her interview further supports the importance of rationale-building (DuBay et al., 2018).

### 4.1. Clinical Implications

Results provide valuable insights into culturally adapting NBDI coaching programs for Spanish-speaking families. This is critical as family involvement and coaching are key elements of NDBI (Minjarez et al., 2020a) but families receiving early intervention services may fail to implement clinician recommendations or discontinue services when there are cultural mismatches (DuBay et al., 2018; Long et al., 2015; Sands et al., 2023). First, experimental and qualitative findings further support utilizing the EVF framework (Bernal et al., 1995). This approach can also include joint planning and rationale building with families (DuBay et al., 2018; Gevarter et al., 2021). Additionally, it may be useful to conduct on-going check-ins with family members to make individualized adaptations throughout the coaching process. Although clinicians should continue to identify and incorporate a child’s preferred play items and activities into NDBI, gathering information about other activities of daily living (e.g., mealtimes and community outings), or play routines in which adults are most interactive/comfortable may serve as a cultural adaptation. Findings also suggest that clinicians working with Hispanic families may need to provide rationales for using contextually relevant spoken models. In this study, all but one adult participant (Raquel) had lower rates of response techniques in comparison to elicitation (like Gallegos et al., 2024). Participants often used words of praise and affirmations rather than providing a contextually relevant spoken model, which is consistent with descriptive research examining how Spanish-speaking families respond to children (Peredo et al., 2020). Another impacting factor is that the of the feedback the lead instructor provided to students regarding this technique may have been influenced by her receptive Spanish knowledge. It may be helpful to use a strengths-based approach to teach caregivers to expand on these words and phrases with more contextual descriptions (e.g., *that’s it, you want a car!*). Additional efforts to enhance coaching surrounding this technique (e.g., providing more models and visuals, teaching self-reflection, and giving more direct feedback) may also be beneficial. 

Clinically, NDBI coaching should also include a discussion of when to appropriately reduce temptations or discontinue a session versus when to try alternative approaches (e.g., changing materials, varying a temptation, or following the child’s lead when they do not show interest in a temptation). Experimental data, qualitative findings, and anecdotal observations suggest that participants’ use of communication techniques were impacted by child setting events (e.g., illness or difficulty with transition) or material satiation (e.g., showing less interest in some items). These findings are supported by Gallegos et al. (2024) and Gevarter et al. (2021). Although decreasing temptation frequency during some sessions may lead to more gradual increases in child communication, it would also reflect a more child-led and neurodiversity-affirming approach. However, novice clinicians and caregivers may need to be taught when to decrease temptations versus when to use establishing operations to increase motivation.

Finally, findings also suggest important implications for graduate programs seeking to better support bilingual students and Spanish-speaking families. Notably, this study provides a novel approach to addressing both systematic access barriers for Spanish-speaking families seeking early intervention autism services (Guerrero & Sobotka, 2022; Magaña et al., 2013; Zuckerman et al., 2017), as well as SLP-reported clinical education training barriers surrounding bilingualism (Martinez-Nieto & Gilbertson, 2025; McKenna et al., 2025). Implementing a cascading coaching model within a university program allows for flexibility in utilizing EVF components, which participants identified as contributing to their positive learning experience. For example, a university clinic may offer increased accessibility for families due to factors such as flexible scheduling and inclusion of additional family members. Additionally, as qualitative findings suggest, student clinicians may have more time to provide dedicated, individualized, culturally relevant interventions than school-based SLPs. Cascading coaching models can also provide bilingual graduate students with more opportunities to be supervised when administering interventions in another language. This is particularly beneficial in a career field with a limited amount of bilingual service providers (ASHA, 2025). Considerations of fit between the language skills of a supervisor, graduate clinician, child, and caregiver are, however, necessary. For instance, although the lead instructor in this study did not have the Spanish skills needed to coach families, her receptive and expressive language knowledge informed her ability to make recommendations for minimally verbal autistic children. Although student clinicians did suggest that more support for translation would be helpful, their overall positive feedback regarding the program and the ability to work directly with Spanish-speaking families suggests clear benefits to their overall learning, even when a fluent Spanish-speaking supervisor was not available. To offset the burden placed on students to use their own resources for translation, graduate programs should provide support (e.g., funding for translation dictionaries; courses focused on clinical vocabulary in commonly spoken languages). Graduate programs could also emulate San Diego State University’s *Habla con Confianza* program which provides a safe space for students to share resources, build community, and practice Spanish (Acosta et al., 2024),

### 4.2. Limitations and Future Research Directions 

Several limitations suggest important future directions. Although the design allowed for four demonstrations of the effect, because there were only two tiers in each implementation of the design (not allowing for the demonstration of effects across three distinct points in time), and baseline phases did not include 5 data points, the design would only meet What Works Clearinghouse (2022) standards for multiple baseline/probes designs with reservations. However, it is important to note that there is some research suggesting that two tiers may be sufficient in multiple baseline designs (Lanovaz & Turgeon, 2020). It has also been suggested that the five data point requirement may present implementation challenges that limit the ability to conduct research in real-world contexts (Harris et al., 2019). In this case, the time constraints of the summer clinic (and associated funding used to support the program) prevented longer baseline collection periods and the ability to extend cascading coaching across a third tier for each participant group. Still, as this is a preliminary investigation, more research is needed to demonstrate reliable intervention effects across participants and contexts. Extensions of this study could explore whether student clinicians generalize coaching techniques to additional caregivers or contexts with the same target child, or to other families and children.

The small sample size of this study also limits generalizability. However, results align with prior research using similar coaching approaches with English-speaking Latino participants (Gallegos et al., 2024; Gevarter et al., 2021). Findings also bolster similarly positive outcomes for caregiver behaviors reported in NDBI studies with Spanish-speaking families (Lopez, 2024; Meadan et al., 2020; Pickard et al., 2024). More research is needed with Spanish-speaking families, and families who speak other non-English languages. Future studies should also investigate how NDBI can be culturally adapted for other underrepresented groups, children with varying language abilities, and across other novice clinician groups (e.g., registered behavior technicians). Additionally, while this study incorporated social validity measures (semi-structured interviews) and a form of generalization (different adult participants using varied materials), no maintenance or additional generalization data were collected. The short duration was noted as a limitation by adult participants, and one suggested a debrief session would be beneficial. Future research could examine caregivers’ continued use of techniques (and more advanced skills) in home. Although experimental child data are not included (to be reported in a related manuscript), improvements in child communication are supported by interviews. Findings could be bolstered via additional social validity measures such as novice viewer ratings of changes in adult and child behaviors.

Another limitation involved the fact that the interaction component of response techniques was broadly defined (i.e., the adult provided time for the child to interact or play prior to initiating another temptation). This response technique was added to address a limitation noted by Gallegos et al. (2024) in which researchers observed that some participants overly prioritized creating temptations rather than allowing ample time to engage in play (resulting in less naturalistic sessions). The current definition of interaction still did not incorporate measures of social engagement. Interestingly, however, caregiver interviews suggested that they saw positive changes in their interactions with children during the study. Refining the definition of interaction and incorporating more instruction and coaching regarding social engagement would be appropriate. Positive relationship changes between caregivers and children, which were suggested by qualitative data, could also be confirmed via observation. Further qualitative studies could more deeply explore how participants view the relationship between changes to their child’s communication and overall improved social interactions as well as how these changes impact adult learning and buy-in.

The addition of an AAC device for Zandra after baseline is also a limitation. Although this addition was the most client-centered and ethical approach based on needs observed at baseline, future research should consider conducting a pre-baseline observation to decide if aided AAC would be beneficial. Additional limitations suggest a need for procedural adaptations. First, in comparison to a similar study involving student clinicians (Gallegos et al., 2024), this study showed greater variability in student coaching fidelity. Although fidelity check-ins helped resolve this issue, more front ending of supports may be needed. For instance, student clinicians likely experienced increased cognitive demands associated with learning methods in English and having to implement them in Spanish (supported by qualitative findings). In addition to providing more translation support, researchers could implement more frequent instructor check-ins, teach students to conduct self-reflection, or use an implementation science framework to gain suggestions for improvement before and during the study (Olswang & Prelock, 2015). Self-reflection could also be used to support adult participants’ addressing negative feelings related to learning new skills in a research setting. Having post-baseline discussions to acknowledge which temptations and skills participants were already using, if any, could also help address this issue. Student clinicians may also benefit from direct feedback on the quality of their coaching techniques. As fidelity assessments in the current study only examined whether students applied the coaching steps, it is unclear whether there were qualitative differences between lead instructor coaching and student-clinician coaching. For instance, it is possible the lead instructor provided more rich or in-depth constructive feedback on NDBI techniques. Although it is important to note that caregivers still showed improvements in their rate of technique use with student-clinician coaching, it is unclear if outcomes were influenced by the overall coaching quality. Thus, future research should aim to include measures that assess the quality of both lead instructor and student-clinician coaching (Gallegos et al., 2024).

## 5. Conclusions

The brief cascading coaching model led to an effective transfer of skills across invested partners with varying levels of NDBI expertise (e.g., BCBA-D, graduate student clinicians, and caregivers of autistic children). This transfer of skills also occurred across languages, from English to Spanish. The qualitative sub-theme of *proactive and responsive individualized, culturally relevant adult and child learning* that was identified as an area of effectiveness across participants supported the fact that EVF cultural adaptations contributed to the overall positive changes in adult participant behaviors demonstrated in experimental findings. Although there are areas for improvement (e.g., more language supports), the study’s social validity was further enhanced by qualitative findings indicating additional benefits across adult perspectives and learning, improved child communication, and caregiver-child relationships. Due to study limitations and the small sample size, findings are preliminary and must be replicated. However, the study has important practice implications as it provides an approach to reducing barriers surrounding (a) non-English speaking caregivers’ access to NDBI coaching and early autism intervention services, (b) bilingual graduate students’ ability to gain experience working with CLD families. As BCBA and SLP training programs recruit more bilingual students, incorporation of culturally adapted evidence-based interventions and caregiver coaching within flexible university programs can enhance the learning experience of student clinicians, while also increasing accessibility to services for CLD families who have autistic children. 

## Figures and Tables

**Table 1:** Child Participant Characteristics.

Child	Age	Ethnicity and Gender	Diagnostic Status	CARS-2 or GARS-3 Score	VABS-III Age Equivalents	Communication Matrix Summary
Triad 1 Gustavo	2;11	Hispanic male	Independent ASD diagnosis	34.5 on CARS-2 (mild-to- moderate ASD)	Expressive: 0;11 Receptive: 1;0	Level 3 (unconventional communication) and Level 4 (conventional gestures) with emerging Level 6 (signs and words)
Triad 2 Zandra	4;3	Hispanic female	Independent ASD diagnosis	94 on GARS 3 (Level 2 requiring substantial supports)	Expressive: 1;0 Receptive: 0;6	Level 3 (unconventional communication)

**Table 2:** Adult participant characteristics.

Adult	Age	Ethnicity and Gender	Highest Education Level	Job or Degree Program
Triad 1 Martha (Gustavo’s grandmother)	72;3	Hispanic female	Middle School	Retired
Triad 1 Nadia (Gustavo’s clinician)	22;5	Hispanic female	BA; Master’s degree in progress	Speech-language pathology graduate student
Triad 2 Fernanda (Zandra’s mother)	41;8	Hispanic female	Postgraduate degree in dentistry	Stay-at-home parent
Triad 2 Raquel (Zandra’s clinician)	28;5	Hispanic female	BA; Master’s degree in progress	Speech-language pathology graduate student

**Table 3:** EVF Framework Components.

Dimensions	Description	Example(s) of Cultural and Linguistic Adaptations Used
Language	Use of culturally appropriate language when providing intervention	Recruitment flyers, coaching, and intervention materials provided in the caregiver’s home language; Bilingual student clinicians provided caregiver coaching.
Persons	Alignment of race, ethnicity, culture, and language between clinicians and families	Bilingual, bicultural, Spanish-speaking Latina student clinicians provided caregiver coaching
Metaphors	Use of common symbols and culturally relevant concepts	Training materials were reviewed by heritage speakers to ensure clear and culturally relevant language
Content	Incorporation of cultural values, customs, and traditions within intervention content	Inclusion of additional family members in the program (e.g., Grandmother was interventionist in Triad 1; Mom and dad provided input for Triad 1 during screening, the child’s sister in Triad 2 attended most play sessions alongside the child) to incorporate *familismo* (cultural concept emphasizing shared family support and decision making); RAISD parent report used to select culturally appropriate materials and routines.
Concepts	Culturally and contextually relevant intervention methods	An individualized adaptation for Triad 2 to include mealtime after the mother indicated this was when she interacted most with her child
Goals	Ensuring treatment goals align with family priorities and cultural values	Joint planning with families was incorporated into coaching; Researchers had a collaborative discussion with the mother in Triad 2 to determine whether introducing and use of an AAC device aligned family goals.
Methods	Integration of cultural knowledge into treatment implementation and procedures	Routines selected based on parent report of child preferences; Joint planning with families incorporated into coaching; Expectations for wait time and modeling language were simplified based on prior research indicating Hispanic families may have challenges with some of these techniques due to cultural mismatch (DuBay et al., 2018; Gallegos et al., 2024; Gevarter et al., 2021; Peredo et al., 2020)
Context	Addressing social, economic, and political factors to support cultural sensitivity	Monetary incentives provided; Collaboration between caregivers, researchers and University clinic to assist navigating insurance claims to cover travel and lodging costs to attend program for Triad 2

**Table 4:** Adult Targeted Temptations + Child-specific targets.

	Adult Targeted Temptations	Example of Child Responses
Triad 1 Student Clinician Nadia’s targets for Gustavo	Interrupting routines + carrier phrase (1, 2, _)	Place third finger up (representing three), sign MORE, say *tres*
	Items that require assistance	Sign or say *ayuda*, hand item to adult
	Hiding or concealing items	Sign or say *ayuda*
Triad 1 Gustavo’s Caregiver Martha’s targets	Interrupting routines + carrier phrase (1, 2, _)	Place third finger up (representing three), sign MORE, say *tres*
	Giving choices	Touch item, point, or reach
	Items that require assistance	Sign or say *ayuda*, hand item to adult
Triad 2 Student Clinician Raquel’s targets for Zandra	Inadequate portions	Aided (symbols for items), persistent vocalization, guiding adult’s hand
	Interrupting routines	Aided AAC (symbols for activity), persistent vocalization, guiding adult’s hand
	Hiding or concealing items	Aided AAC (symbols for item or activity), persistent vocalization, guiding adult’s hand
Triad 2 Zandra’s Mother Fernanda’s targets	Inadequate portions	Aided AAC (symbols for items), persistent vocalization, guiding adult’s hand
	Interrupting routines	Aided AAC (symbols for activity) persistent vocalization, guiding adult’s hand
	Items requiring assistance	Aided AAC (symbols for activity), persistent vocalization, guiding adult’s hand

**Table 5:** A Priori Categories.

	Learning	Perspective	Behavior	Relationships
Areas for Improvement	Increase learning opportunities (CG & SC) Provide more Spanish support (SC)	Address negative feelings related to learning new skills in research setting (CG & SC)	Not applicable	Not applicable
Effectiveness	Positive learning experience (CG & SC) Program’s accessibility (CG) Dedicated time (CG & SC) Proactive and responsive individualized, culturally relevant adult and child learning (CG & SC) Cascade Model (CG & SC)	Benefits of NDBI (CG & SC)	Child Communication Improvement (CG & SC). Increase in adult participants’ use of NDBI strategies (CG & SC)	Positive change in relationship between caregiver and child (CG)

*Note.* CG = Caregiver, SC = Student Clinician.

## Data Availability

Original data tables for the single case experimental design are available upon request to the corresponding author. Full interview transcriptions are unavailable due to privacy restrictions.

## Notes

1 The authors are intentionally switching between identity first language and person first language to acknowledge the diverse perspectives on language preferences.

2 While the terms *Hispanic*, *Latino*, and *Latina* are used in this work to reflect the self-identification of the adult participants, the author acknowledges the evolving and complex nature of racial, ethnic, and cultural terminology, and recognizes that alternative terms such as *Latinx*, *Latine*, and *Latin@* are also used in various contexts.

## Supplementary Materials

The following supporting information can be downloaded at https://www.mdpi.com/article/10.3390/bs15091292/s1, Figure S1: Instructional Visual Aid in Spanish and English; Figure S2: Example of Blank Activity Planner in Spanish and English; Figure S3: Coaching Checklist for Sessions 1–3 and Sessions 4–6; Figure S4: Example of Proloquo2Go AAC application page for one preferred set of items; Figure S5: Student Interview Questions; Figure S6: Caregiver Interview Questions.
