# Supplementary material for: Coaching Bilingual Speech-Language Student Clinicians and Spanish-Speaking Caregivers to Use Culturally Adapted NDBI Techniques with Autistic Preschoolers
Source: Behav Sci (Basel). 2025 Sep 22;15(9):1292. doi: 10.3390/bs15091292 (PMC12467700; doi:10.3390/bs15091292)
Supplement: Supplementary file 1 [file behavsci-15-01292-s001.zip › behavsci-3760776-supplementary.pdf]

Figure S1. Instructional Visual Aid in Spanish and English

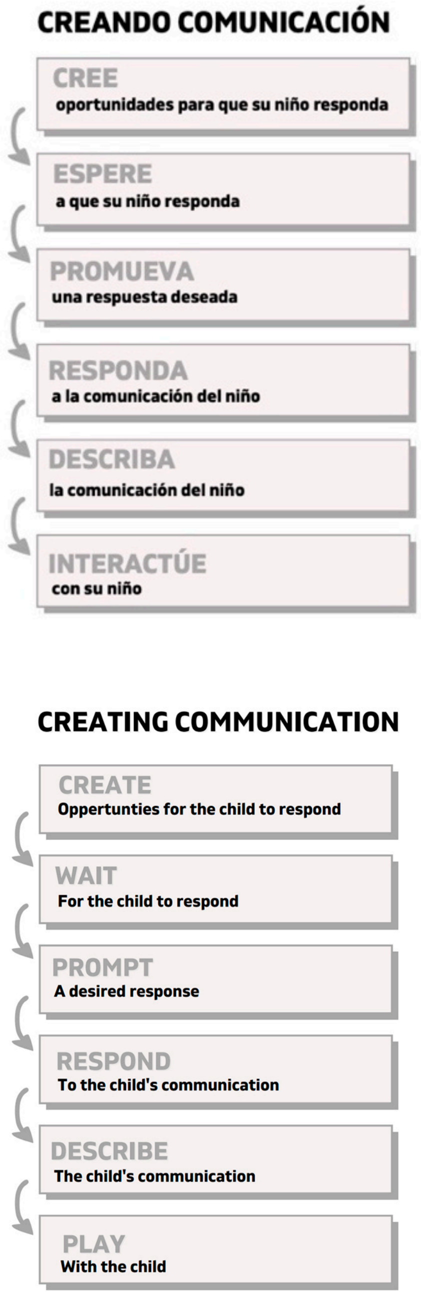

Figure S2. Example of Blank Activity Planner in Spanish and English

Niño/a: ________________ Estudiante Graduado: ______________ Padre / Familiar: _______________

Oportunidad de Comunicación:	Actividades/ Rutinas:	Tipos de Respuestas:	Tiempo de Espera:	Apuntar:	Responda y Describa:	Interactúe:
	Variaciones		A lo Menos 3s		Responda:<br>Describa:	Maneras de jugar
	Variaciones		A lo Menos 3s		Responda:<br>Describa:	Maneras de jugar
	Variaciones		A lo Menos 3s		Responda:<br>Describa:	Maneras de jugar

Target Temptation	Activities/Routines	Types of Responses	Wait Time	Prompt	Respond & Describe	Play
	Variations		At least 3s		Respond:<br>Describe:	Ways to engage/play
	Variations		At least 3s		Respond:<br>Describe:	Ways to engage/play
	Variations		At least 3s		Respond:<br>Describe:	Ways to engage/play

Figure S3. Coaching Checklist for Sessions 1 – 3 and Sessions 4-6

Coaching Checklist Sessions 1-3:

Sessions 1-3:

- ☐ Introduce and model one communication temptation and strategies needed to complete communication turn
- ☐ Fill in activity planner jointly
- ☐ Visual aid available
- ☐ Ask for questions

All sessions:

- ☐ 15-minute play session
- ☐ Bag of child's preferred items available
- ☐ Used verbal cues and assistance as needed

Provide Feedback (at least 3 strategies):

- ☐ Create
- ☐ Wait
- ☐ Prompt
- ☐ Respond
- ☐ Describe
- ☐ Play

Coaching Checklist Sessions 4-6

Sessions 4-6:

- ☐ Activity planner available
- ☐ Visual aid available
- ☐ Ask for questions
- ☐ Model strategies if needed

All sessions:

- ☐ 15-minute play session
- ☐ Bag of child's preferred items available
- ☐ Used verbal cues and assistance as needed

Provide Feedback (at least 3 strategies):

- ☐ Create
- ☐ Wait
- ☐ Prompt
- ☐ Respond
- ☐ Describe
- ☐ Play

Figure S4. Example of Proloquo2Go AAC application page for one preferred set of items

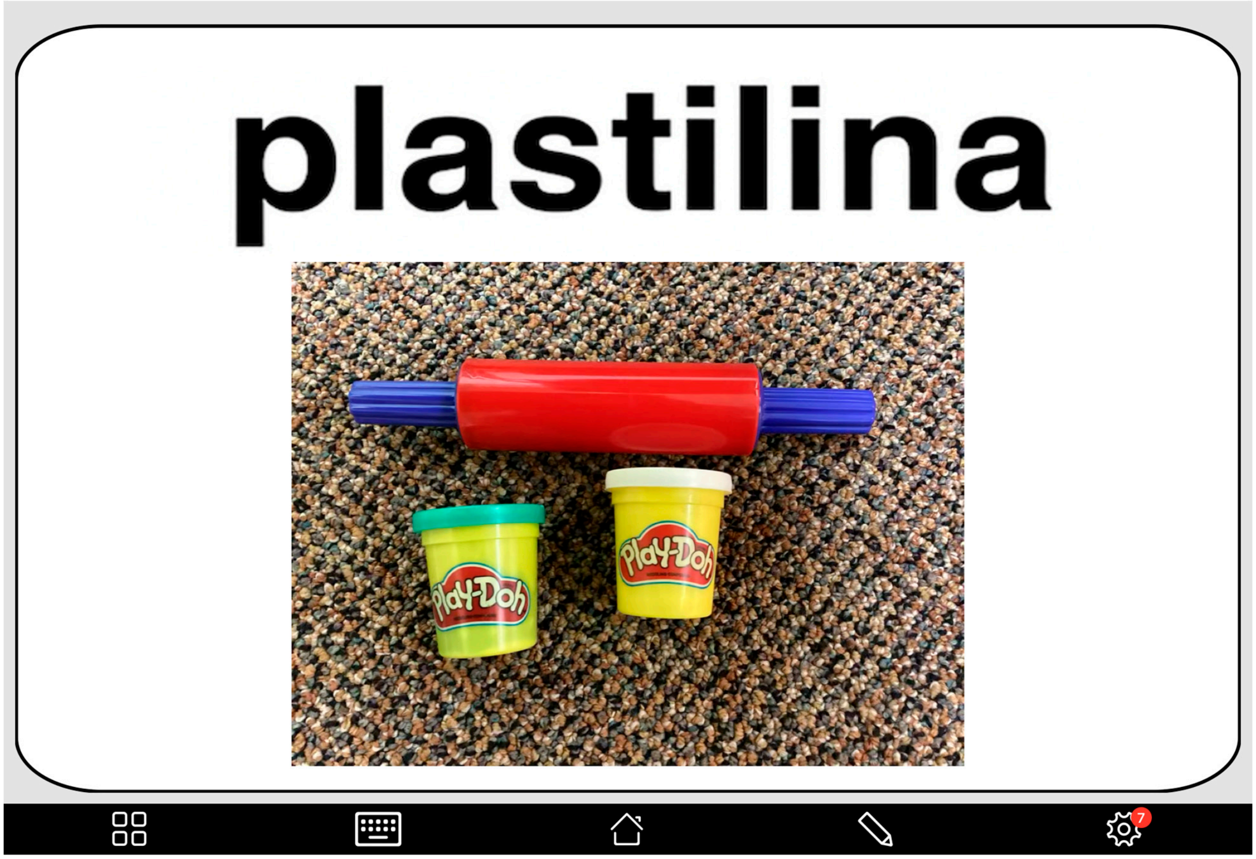

Figure S5. Student Interview Questions

1. Please describe the target child's communication skills when you first started to work with them and how it compared to their social communication skills by the end of the study.
2. What was your experience like during this study?
    a. Were there any particularly useful aspects?
    b. Were there any particularly difficult / hard to implement / less useful aspects?
    c. Were there any aspects of the program that you would suggest changing?
    d. How was your experience learning the strategies in English and then teaching that information in Spanish to parents / caregivers?
    e. Probe for cultural considerations / values / primary language of the parent / caregiver
3. Tell me about your experience working with the parent / caregiver
    a. What was this like in comparison to working with other families (e.g., English speakers)
    b. Elaborate on positive elements
    c. Elaborate on any challenges

Figure S6. Caregiver Interview Questions

1. Por favor dígame qué le pareció este programa. (Please tell me what you thought of this program)

2. ¿Hubo algo en particular que fue de ayuda para usted en este programa? (Was there anything in particular that was helpful to you during this program?)

3. ¿Hubo algo en particular que fue difícil para usted en este programa? (Was there anything in particular that was difficult for you during this program?
    a. ¿Hay algo que usted cambiaría en este programa? ¿Tiene sugerencias? (Is there anything you would change about this program? Do you have any suggestions?)
    b. ¿Puede dar su opinión sobre cómo este programa tuvo en cuenta o no sus valores culturales y su idioma? (Can you give your opinion on how this program did or did not take into account your cultural values and language?)

4. Por favor, describa las habilidades de comunicación de su hija/nieto antes y después del programa.(Please describe your daughter/grandson's communication skills before and after the program.

5. Por favor, describa cómo interactuaba con su (hija/nieto) antes del programa y cómo interactúa con ella ahora. (Please describe how you interacted with your daughter/grandson before the program and how you interact with them now).
